# The impact of co-occurring chronic pain and mental health symptoms on adolescent functioning, a cross-sectional survey

**DOI:** 10.1186/s40359-024-02126-5

**Published:** 2024-11-06

**Authors:** Sharon Bateman, Abbie Jordan, Melanie Noel, Maria Loades, Line Caes

**Affiliations:** 1 Department of Psychology, University of Bath, Claverton Down, BA2 7AY Bath UK; 2 Centre for Pain Research, University of Bath, Claverton Down, Bath BA2 7AY UK; 3 Department of Psychology, University of Calgary, 2500 University Dr, NW Calgary, AB T2N 1N4 Canada; 4 Division of Psychology, Faculty of Natural Sciences, University of Stirling, Stirling, FK9 4LA UK

**Keywords:** Adolescence, Pain, Functioning, Mental health, Co-occurring

## Abstract

Adolescents who experience poor mental health may develop chronic pain. Similarly, those with chronic pain may develop mental health symptoms. Little is known concerning how these co-occurring symptoms are associated with adolescent functioning. Our online cross-sectional quantitative study compared the specific nature of challenges to psychosocial functioning, across 4 groups: (1) adolescents with co-occurring chronic pain and mental health symptoms; (2) adolescents who only experience chronic pain symptoms; (3) adolescents who only experience mental health symptoms, and (4) adolescents who do not report either symptom. Participants completed self-report questionnaires assessing pain experiences, mental health symptoms and psychosocial functioning. After controlling for pain intensity, findings revealed significant differences, in physical, social, and family functioning across all groups. Significantly worse physical functioning was reported by adolescents with co-occurring pain and mental health symptoms and those with pain-only symptoms compared with adolescents with mental health-only symptoms and those without symptoms. Social functioning was significantly worse for adolescents who experienced co-occurring chronic pain and mental health symptoms compared with adolescents who experienced pain-only symptoms, and those with no symptoms. Significantly worse family functioning was reported by adolescents with mental health-only symptoms compared with adolescents reporting pain-only symptoms and no symptoms. Results suggest the combined impact of pain and mental health symptoms mainly influences adolescent social functioning. Future research is needed to develop standardised assessment and treatment plans to facilitate a thorough understanding of symptoms presented by adolescents who experience co-occurring pain and mental health symptoms.

This study identifies that the combined impact of pain and mental health symptoms influences adolescent social functioning when compared to adolescents who experience pain-only or those without symptoms. Research is needed to develop standardised assessment and treatment plans to fully understand the symptoms presented by adolescents with co-occurring symptoms.

**Supplementary Information:**

The online version contains supplementary material available at 10.1186/s40359-024-02126-5.

## Introduction

Adolescence is characterised by a period of physical, psychological, and biological changes [1–3]. An increasing number of adolescents develop mental health symptoms during this developmental period [4, 5]. Worldwide figures indicate that 10–20% of adolescents experience mental health disorders, with anxiety and depression being the most prevalent diagnosed conditions in this population [6]. In the UK, 18% of young people aged 7–16 and 22% of those aged 17–24 years reported a probable mental health disorder [7]. However, this figure may be conservative as there are multiple reasons why adolescents may not seek support for poor mental health symptoms, such as limited personal knowledge of mental health in addition to a lack of understanding surrounding the seriousness of their symptoms and/or where to seek help [8].

Chronic pain is common in adolescents and associated with increased mental health problems. Rates of chronic pain in adolescents are varied, recent findings reporting that 44.2% of adolescents experience weekly chronic pain over the previous 6 months, with rates increasing in adolescence and in females [9]. Chronic pain is associated with poor mental health symptoms in young people i.e. anxiety, depression, and behavioural disorders [10–13]. Indeed, a recent retrospective analysis of self-report questionnaire data, collected at an outpatient pain clinic, (*n* = 155 adolescents, 13–18 years) found that 16% of adolescents living with chronic pain also experienced co-occurring mental health symptoms (depression) [14], although this analyses was based on brief screening measures with limited validity.

Many adolescents who experience chronic pain and increased levels of mental health symptoms also report impaired physical, social, and emotional functioning. For example, higher levels of self-reported depressive and anxiety symptoms in adolescents who experience chronic migraine are associated with poorer physical, social and emotional functioning than adolescents with episodic migraine or no pain [15]. However, the relationship between chronic pain, functioning, and mental health symptoms in adolescents is often complex and poorly understood. To illustrate, chronic pain research is often conducted with adolescents who experience chronic pain and elevated symptoms of depression and anxiety, which are a common response to the experience of pain [16, 17]. In contrast, there is a dearth of research to address how a broader range of mental health symptoms such as those associated with eating disorders, attention deficit hyperactivity disorder (ADHD) and self-harm, co-occur with chronic pain. Furthermore, research exploring the association between chronic pain and mental health symptoms rarely recruit comparison groups of adolescents who experience mental health symptoms without pain, or a control group of adolescents who neither report pain nor mental health symptoms. Subsequently, less is known about the individual versus the additive impact of these symptom groups on adolescents’ psychosocial functioning which is important in terms of informing individualised interdisciplinary treatment plans.

This exploratory study aimed to bridge the gap in knowledge between adolescent groups with and without symptoms to identify the specific nature of any challenges to psychosocial functioning, i.e., developmental social, family, physical and peer relationships, faced by adolescents who experience: (1) co-occurring chronic pain and mental health symptoms and compare with (2) adolescents who experience chronic pain-only or with (3) mental health symptoms only and with (4) those adolescents who do not report either pain or mental health symptoms.

## Methods

This cross-sectional self-report questionnaire-based online survey was approved by the UK National Health Service Ethics Committee (Approval number: 19/YH/0182).

### Participants

Participants were recruited for a wider study made available on the Open Science Framework (https://osf.io/msd47) between October 2019 and August 2020 at national specialist chronic pain clinics, secondary schools within the UK and via social media. The current study is focussed only on the domain of functioning of adolescent participants, who experience symptoms of mental health, chronic pain, co-occurring mental health and pain or no symptoms. Thus, we used only the assessment measures relevant to adolescent functioning. Our wider study collected data on an expansive variety of mental health and chronic pain symptoms, along with parental reports on their own and their child’s symptoms. A description of the assessment measures used in the wider study can be found on the Open Science framework detailed above. Consequently, the sample size for this study was determined based on an à priori calculation for the larger study, targeting 250 participants to detect a small effect size at significance level 0.1 and a power of 0.80. An estimated loss of 50 participants was anticipated due to expected attrition. Due to Covid 19 our recruitment methods were amended and more diverse than originally planned. We had approval to recruit through local Child and Adolescent Mental Health Services (CAMHS), although no participants came forward form these centres. Eligibility criteria for participation were consistent for all participants. However, the targeted recruitment information was tailored to specific contexts to ensure clarity. Specifically, recruitment efforts aimed at adolescents from schools or universities who did not experience pain or mental health symptoms highlighted their eligibility to participate even if they did not attend clinics or experience such symptoms. The inclusion criteria were as follows: (1) being aged 11–19 years; (2) experience pain or mental health symptoms, for those in the symptomatic groups; (3) being a pupil in school/college or university for those in the non-symptoms group; (4) have a computer and internet access or be happy to receive paper copies of the questionnaires; (5) have the cognitive competency to be able to complete study tasks; (6) Be able to read, write, speak English to the level required to complete study recruitment tasks.

### Procedure

For recruitment in clinical settings, potential participants were approached by the researcher or their clinician, prior to or following their clinic appointment, and provided with information about the current study. Those interested in participating were given an information leaflet with an email address and a QR code, which provided an online link through Qualtrics [18], a secure online survey platform. Alternatively, the potential participant provided the researcher with their email address so that the Qualtrics study link could be emailed to them. For recruitment through schools, potential participants were either sent written information about the study, through inclusion of the advertisement and QR code of the study in the school newsletter or were provided with information leaflets and a QR code after an in-person presentation by the lead author SB. Recruitment was also conducted online via chronic pain and mental health charities and social media platforms, such as Twitter, Facebook, and Instagram. The online sample was recruited using a post on social media which provided a link to the study overview. For those wishing to participate, a further link to the online platform was available in this overview page. Participants were also asked to share the link with eligible friends that might like to take part, these participants followed a link to the study overview and another link if they wished to take part. The majority of participants were recruited via social media (*n* = 49 (35.8%)), followed by specialist chronic pain clinics (*n* = 33 (24.0%)), school, college, or university (*n* = 28 (20.4%)), and snowballing (*n* = 27 (19.7%)).

On accessing the online portal, all potential participants were asked to specify their age to enable the age-appropriate participant information to be displayed, (selection categories: youth 11–15; youth 16–19, or parents required to provide consent for youth aged 11–15). The online information was designed in two parts with part one providing a brief outline of the study. Following the initial confirmation of interest and eligibility, adolescents were directed to part two, a more detailed participant information page and the relevant age-appropriate consent/assent page. All adolescent participants were asked to provide informed consent (16 years and above) or assent (under 16 years) to confirm their willingness to take part in the study. Adolescents under 16 years of age were required to provide contact information for a parent/caregiver to allow parental consent to be sought prior to their participation in the study. Confirmation of parental consent was checked by the lead author. Once participants confirmed their eligibility to take part, they were able to access the online questionnaire and choose their own unique identification code which consisted of their initials and date of birth, which would enable the removal of their response should they change their mind about participation. The questionnaire included a range of demographic questions and psychometrically robust assessment measures addressing a range of domains. Whilst participants were able to elect to skip survey questions, if multiple questions in the same assessment measure were missed or participants did not complete the study, a unique code was sent to them inquiring whether the omissions were in error and inviting them to complete the questions if they wished. A maximum of two reminder emails were sent. Following screening for fraudulent participation, each participant received an online shopping gift card to the value of £10 (GBP) to thank them for their participation in the study.

### Measures

The adolescent survey comprised demographic questions and a battery of psychometrically-sound self-report measures. Domains of assessed functioning included: developmental, social, family, and physical functioning, peer relationships in addition to characteristics of pain and mental health symptoms.

#### Demographic questions

A thorough bank of questions were developed to ascertain key participant variables including their experience of chronic pain and mental health symptoms, age, gender, pain symptoms, mental health symptoms, duration of any symptoms, if symptoms were formally diagnosed or if any treatment had been requested or received.

An example of the questions asked:

Have you ever, or are you currently experiencing pain or mental health symptoms that have lasted for three months or longer?

Possible answers included:

- Yes, pain symptoms.
- Yes, mental health symptoms.
- Yes, both pain and mental health symptoms.
- No pain or mental health symptoms.

Depending on the participants response they were directed to further relevant questions, for example:

Please describe your mental health symptoms (a free text box was provided for individual responses).

See full demographic questionnaire and available responses in supplementary material 1. The participant’s response to the question; ‘have you ever or are you currently experiencing pain or mental health symptoms that have lasted for three months or longer’, allowed us to allocate participants into one of four groups depending on their chosen response from the available answers of: (1) pain symptoms only; (2) mental health symptoms only; (3) both pain and mental health symptoms; or (4) no pain or mental health symptoms. These groupings were then used as our primary symptom variable in our analysis.

### Assessment measures

*The Bath Adolescent Pain Questionnaire* (BAPQ: [19]) is a validated and reliable measure that comprises seven subscales, that measure the impact of pain on social functioning, physical functioning, depression, general anxiety, pain specific anxiety, family functioning and development. All subscales demonstrate good reliability with established measures [19] and internal consistency for the subscales [20]. The 11-item Development and 9-item Social Functioning subscales were used for this study to assess the adolescent’s perceptions of how they function developmentally and socially. The Developmental subscale uses a 5-point scale for participants to indicate their perceived progress of the task in comparison with their peers, ranging from 0 (very behind) to 4 (very ahead). The Social Functioning subscale uses a 5-point frequency response scale, ranging from 0 (never) to 4 (always). For example, ‘I have difficulty spending time with groups of people’. The BAPQ social functioning and developmental subscales used in this study are appropriate for completion by all the adolescents in our sample as neither subscale references pain in the subscale instructions and the measure overall makes it clear that pain can impact adolescents’ lives in different ways. The instructions state “There are many possible ways that pain can affect the lives of young people. Below are some statements that may or may not apply to you”. Such an instruction makes it clear that items may not be applicable to some participants. Higher scores for each subscale indicate more impaired functioning. In the current study the Cronbach alpha coefficient was recorded as 0.76 for the Developmental subscale and 0.86 for the Social Functioning subscale.

*The Patient Reported Outcomes Measurement Information System* (PROMIS Pediatric Profile v2.0 Profile-25: [21–23]) is a 25 item self-report measure that indicates the individual’s satisfaction with their quality of life. It was developed using rigorous qualitative and quantitative methods [24] and contains seven subscales of physical function mobility, anxiety, depression, peer relationships, fatigue, pain interference and pain intensity. Good internal consistency and construct validity have been demonstrated across the measures for children [25]. The subscales of Physical Functioning Mobility, Peer Relationships and Pain Intensity were used in this study to assess adolescent physical functioning, friendships and how they rated their pain intensity. Participants were asked to answer these measures while thinking about the past 7 days. A 5-point Likert scale is used to assess each item. The Physical Functioning Mobility subscale assesses the level of physical function that participants experienced with responses ranging from 5 (with no trouble) to 1 (not able to). The Peer Relationships subscale enquired about friendships, with responses ranging from 1 (never) to 5 (almost always). Lower scores indicate less satisfaction across both physical functioning and peer relationships. Total raw scores for physical functioning mobility and peer relationships were converted into standardised T-scores for the analysis M = 50, SD = 10. 0. T-scores are standardised to a USA general population and conversion tables are available from www.healthmeasures.net. The current study revealed good reliability, with Cronbach alpha coefficients of 0.89 for physical functioning mobility and 0.85 for peer relationships. Pain intensity was recorded using a numerical scale where 0 was equal to no pain, and 10 was equal to the worst pain you can think of.

*The Systemic Clinical Outcome and Routine Evaluation* (SCORE-15: [26]) is a 15 item self-report measure assessing family functioning. SCORE-15 has good internal consistency and construct validity for the total scale [27], and has been validated for clinical use [28]. The SCORE contains three subscales including, Family Strengths, Family Communication and Family Difficulties and a total score. Each item asked the participant to think about their family functioning at that moment and to reflect how the statement described their family with responses assessed using a 5-point Likert scale ranging from 1 (very well) to 5 (not at all). The total scores were used in the analyses of this study, with lower scores indicating better family relationships. In the current study, the Cronbach alpha coefficient was 0.94.

### Data analysis

Data from Qualtrics [18] were downloaded and cleaned using Microsoft Excel prior to being uploaded to SPSS Version 26 [29]. Individual missing scores for the PROMIS physical functioning subscale (*n* = 1) and the BAPQ development subscale (*n* = 1) were replaced with the participant’s mean score for the relevant subscales.

Preliminary testing of the assumptions of normality, linearity, univariate and multivariate outliers, homogeneity of variance-covariance matrices and multicollinearity were conducted (see Table 1), revealing no evidence of multicollinearity thus our data conformed to the assumptions of multivariate analysis of variance analyses (MANOVA) assumptions. Due to unequal or small group sizes of our data set, the more robust Pillia’s Trace was used. Tests of normality revealed significant Kolmogorov-Smirnov statistics for the distribution of scores, however closer inspection of the histograms revealed that scores were reasonably normally distributed for all except the PROMIS physical function mobility measure, consequently this was removed from the MANOVA (see details below). Instead, a separate robust analysis of variance (ANOVA) was carried out on the physical functioning data to allow comparisons across the groups (chronic pain symptoms only, mental health-only symptoms, both chronic pain and mental health symptoms or no symptoms) to be made. Preliminary assumptions were tested and bivariate relationships between the 4 groups and the dependent variables of peer relationships, developmental, social, physical, and family functioning were examined using Pearson product-moment correlation coefficient (r).

Multivariate analysis of variance (MANOVA) was used to examine the differences between the 4 groups on their reports of peer relationships, developmental, social, and family functioning. The Levene’s Test of Equality of Variances for the peer relationships, and the SCORE family functioning variables was violated. Consequently, a more conservative alpha level of 0.01 was set using a Bonferroni adjustment in line with Tabachnick & Fidell [30] to compensate for multiple comparisons and the increased risk of making a Type I error. To further develop the model, ANOVA was used to investigate the interactions to evaluate if there was an effect of age, gender differences or pain intensity. As age and gender did not differ across our groups, only pain intensity was significantly different across the groups and was used as a covariate in follow-up multivariate analysis of covariance analyses (MANCOVA) (peer relationships, developmental, social, and family functioning) and analysis of covariance ANCOVA (functioning). The decision to first conduct a standalone MANOVA facilitated an understanding of the basic multivariate relationships, while incorporating covariates in a subsequent analysis enabled us to control for potential confounding factors, leading to a more nuanced understanding of the relationship between the independent and dependent variables. Noting that our sample was collected for our larger study, to understand the power in this study we used the estimated effect size from our initial MANOVA observations to conduct a post hoc power analysis and concluded that with our given sample our power exceeded 99% at significance level 0.01.

**Table 1:** Pearson’s correlation between adolescent symptom groups and assessment measure variables

Symptoms	Assessment measure	Developmental	Social Functioning	Physical Functioning mobility	Peer Relationships	Family Functioning
Pain (*n* = 20)	Developmental	-	0.51*	− 0.65^**^	− 0.51*	− 0.17
	Social Functioning		-	− 0.50*	− 0.76**	− 0.05
	Physical Functioning Mobility			-	0.25	− 0.20
	Peer Relationships				-	0.29
	Family Functioning					-
Mental health (*n* = 44)	Developmental	-	0.36^*^	− 0.15	− 0.26	− 0.28
	Social Functioning		-	− 0.20	− 0.65**	0.09
	Physical Functioning Mobility			-	0.17	0.09
	Peer Relationships				-	− 0.14
	Family Functioning					-
Both pain and mental health (*n* = 54)	Developmental	-	0.61^**^	− 0.54**	− 0.23	− 0.07
	Social Functioning		-	-23	− 0.65**	0.25
	Physical Functioning Mobility			-	− 0.09	− 0.16
	Peer Relationships				-	− 0.40**
	Family Functioning					-
No symptoms (*n* = 19)	Developmental	-	0.49*	− 0.32	− 0.62**	0.59**
	Social Functioning		-	− 0.45	− 0.79**	0.35
	Physical Functioning Mobility			-	0.38	− 0.30
	Peer Relationships				-	− 0.31
	Family Functioning					-

Note. * Correlation is significant at *p* ≤ .05 (2-tailed), ** Correlation is significant at *p* ≤ .01 (2-tailed)

## Results

### Descriptive statistics

#### Participants

A total of 212 adolescent participants provided consent or assent prior to completing the survey. Of these 212 potential participants, 8 responses were found to be fraudulent, 2 participants under 16 years failed to provide parental consent, 5 were not based in the UK, 1 was over 19 years of age, 1 duplicated their response and 58 failed to complete the full survey. Consequently, the final study sample included 137 adolescents aged between 11 and 19 years (M = 16.07, SD = 1.8) comprising of 114 females (83.2%), 17 males (12.4%) and 6 non-binary or gender fluid (4.4%) adolescents. Participant ethnicity was reported by 136 adolescents, with the majority reporting to be white (95.6%) and a minority reporting mixed race (2.2%), Asian (1.5%) or Black (0.7%). Details of participant characteristics can be found in Table 2.

Many of the adolescents reported having received a diagnosis for their chronic pain (*n* = 49 (35.7%)) and/or their mental health conditions (*n* = 55 (40.1%)). Adolescents who reported chronic pain experienced pain across a wide range of locations, the most frequently reported locations were lower limb pain (*n* = 38, 27.7%), back pain (*n* = 34, 24.8%), headache/migraine (*n* = 30, 21.9%), multisite pain (*n* = 26, 19%) and neck pain (*n* = 24, 17.5%). Most adolescents reported the duration of their chronic pain to be over 5 years (*n* = 25, 18.2%). The most frequently reported chronic pain diagnoses were Complex Regional Pain Syndrome (CRPS, *n* = 22, 16.1%) and chronic pain disorder (*n* = 14, 10.2%). See Table 2 for a full list of reported pain sites and diagnosed conditions. Across the sample the most frequently reported mental health symptoms were anxiety (*n* = 68, 49.6%), depression (*n* = 41, 29.7%), low mood (*n* = 24, 17.5%), panic attacks (*n* = 16, 11.7%) and self-harm (*n* = 10, 7.3%). Most adolescents reported the duration of their mental health symptoms to be over 5 years (*n* = 31, 22.6%). The most prevalent mental health diagnoses reported were anxiety disorder (*n* = 37, 27.0%) and depression (*n* = 24, 17.5%). See Table 2 for a full list of metal health symptoms and diagnosed disorders included. Participants were grouped according to whether they reported either pain symptoms only (*n* = 20), mental health-only symptoms (*n* = 44), co-occurring pain and mental health symptoms (*n* = 54) or no symptoms (i.e., the comparison sample, *n* = 19).

With regard to functioning, we found that participants with co-occurring chronic pain and mental health symptoms reported the poorest level of functioning across the majority of functioning measures. Specifically, they had poorer developmental functioning (higher scores on the BAPQ indicate worse functioning; BAPQ, M = 26.3, SD 6.6), social functioning, (BAPQ, M = 21.9, SD 5.4), and peer relationships (lower scores on the PROMIS indicate less satisfaction; PROMIS, M = 48.2, SD 10.7). The exceptions were adolescents with symptoms of mental health-only, who reported the worst level of family functioning M = 45.3 SD 12.5) and adolescents who experience chronic pain-only, who reported worse physical functioning than the other groups (M = 41.8 SD 8.0). The participants experiencing chronic pain-only reported the highest levels of pain intensity over the past week with a mean average of 7.5 (SD 1.8, range 1–10). Similarly, those participants who experience co-occurring pain and mental health symptoms reported a high average mean of pain intensity (M = 6.5, SD 1.8) compared with those participants experiencing mental health-only (M = 3.1, SD 2.2) or no symptoms M = 2.3, SD 2.4). Further information can be found in Table 3 regarding reported functioning on the different measures across the four study groups.

**Table 2:** Participant demographic, symptom and condition related characteristics

Characteristic	Number (%)
Mean age range 11–19	16.07 SD 1.8
Ethnicity	
White	131 (95.6)
Black	1 (0.7)
Asian	2 (1.5)
Mixed	3 (2.2)
unknown	1 (0.7)
Gender	
Male	17 (12.4)
Female	114 (83.3)
Non-binary	6 (4.4)
Category	
Pain	20 (14.6)
Mental health	45 (32.8)
Both pain and mental health	54 (39.4)
No symptoms	19 (13.8)
Main sites pain experienced in	
Lower limbs	38 (27.7)
Back pain	34 (24.8)
Headache/ migraine	30 (21.9)
Multisite pain	26 (19.0)
Neck pain	24 (17.5)
Upper limbs	23 (16.8)
Abdominal pain	20 (14.6)
Muscle pain	21 (15.3)
Whole body pain	4 (2.9)
Chest pain	2 (1.5)
Other pain symptoms*	21 (15.3)
Duration of chronic pain symptoms	
One year and under	10 (7.3)
1 < 2 years	12 (8.8)
2 < 3 years	15 (10.9)
3 < 4 years	8 (5.8)
4 < 5 years	4 (2.9)
Over 5 years	25 (18.2)
Mental health symptoms experienced	
Anxiety	68 (49.6)
Depression	41 (29.9)
Low mood	24 (17.5)
Panic attacks	16 (11.7)
Self-harm	10 (7.3)
Disordered eating	9 (6.6)
Low self esteem	6 (4.4)
Negative or intrusive thoughts	6 (4.4)
Suicidal thoughts	6 (4.4)
Lack of focus or motivation	6 (4.4)
OCD	5 (3.6)
PTSD	4 (2.9)
Paranoia	4 (2.9)
Anger	4 (2.9)
Fatigue	4 (2.9)
Audio and/or visual hallucinations	3 (2.2)
Fluctuating moods	3 (2.2)
Suppressed emotions or numbness	3 (2.2)
Emotional dysregulation	2 (1.5)
Manic episodes	2 (1.5)
Autistic traits	2 (1.5)
Other mental health symptoms*	18 (13.1)
Duration of mental health symptoms	
One year and under	17 (12.4)
1 < 2 years	11 (8.0)
2 < 3 years	17 (12.4)
3 < 4 years	10 (7.3)
4 < 5 years	8 (5.8)
Over 5 years	31 (22.6)

Note. * Reflects multiple other symptoms and conditions that can found in supplementary material 1

**Table 3:** Mean scores on assessment measures between groups of adolescents with chronic pain-only, mental health only, both chronic pain and mental health symptoms and no symptoms

Adolescent assessment measure	Pain symptoms only *n* = 20			Mental health symptoms only *n* = 44		Both pain and mental health symptoms *n* = 54		No symptoms *n* = 19	
	Range	Mean	SD	Mean	SD	Mean	SD	Mean	SD
BAPQ-Development	0–44	24.4	5.3	23.6	5.9	26.3	6.6	19.0	6.4
BAPQ-Social Functioning	0–36	17.0	5.4	18.4	5.8	21.9	5.4	14.2	5.6
PROMIS-Physical Function^a^	4–20	41.8	8.0	57.6	3.6	43.4	8.5	59.7	2.8
PROMIS-Peer Relationships^a^	4–20	50.0	13.5	50.6	7.3	48.2	10.7	54.4	7.8
SCORE-Family Relationships	15–75	31.8	9.3	45.3	12.5	39.4	13.5	31.3	7.9
PROMIS-Pain Intensity	1–10	7.5	1.8	3.1	2.2	6.5	1.8	2.3	2.4

Note. Higher scores for the BAPQ Development and social measures and for the Score Family Functioning measure indicate more impaired functioning. Higher scores on the PROMIS Physical Function and Peer Relationship measures indicate better functioning and relationships. ^a^ Reflects converted T-scores where a score of 50 is the average in the general population in the USA

### Exploratory analysis for social functioning, developmental functioning, peer relationships and family functioning

A between-within groups MANOVA revealed a statistically significant difference between the groups (pain or mental health-only, both pain and mental health and no symptoms) on the combined dependent variables (social functioning, developmental functioning, peer relationships and family functioning): *F* (12 ,396) = 5.44, *p* = .000; Pillia’s Trace = 0.43; partial eta squared = 0.14. The results for the dependent variables were considered separately and showed that with the exclusion of peer relationships, all differences were statistically significant. Specifically, we found that the participant means (shown in Table 3) for developmental, social and family functioning were statistically different between the groups (developmental functioning: using Bonferroni adjusted alpha level of 0.113, *F* = (3, 133) = 6.77, *p* = .000, partial eta squared = 0.13; social functioning; using Bonferroni adjusted alpha level of 0.176, *F* = (3, 133) = 10.71, *p* = .000, partial eta squared = 0.20; family functioning: using Bonferroni adjusted alpha level of 0.153, *F* = (3, 133) = 9.16, *p* = .000, partial eta squared = 0.17).

Further pairwise comparison investigation revealed the differences lie in those adolescents with co-occurring chronic pain and mental health symptoms who reported significantly worse developmental functioning scores (mean difference = 7.35, SE = 1.65, *p* = .001) and social functioning scores (mean difference = 7.68, SE = 1.48, *p* ≤ .001) when compared to adolescents with no symptoms. Similarly, adolescents with co-occurring symptoms also reported worse social functioning than those with chronic pain-only symptoms (mean difference = 4.89, SE = 1.45, *p* = .006). Lastly, significant differences were also found for family functioning between the mental health-only cohort compared to both the chronic pain-only cohort (mean difference = -13.59, SE = 3.24, *p* = .001) and the no symptom cohort (mean difference 14.08, SE = 3.30, *p =* .001). Specifically, adolescents with mental health symptoms only, reported significantly worse family functioning than adolescents with chronic pain-only or adolescents with no symptoms.

### The influence of pain intensity on functioning

**Fig. 1:**
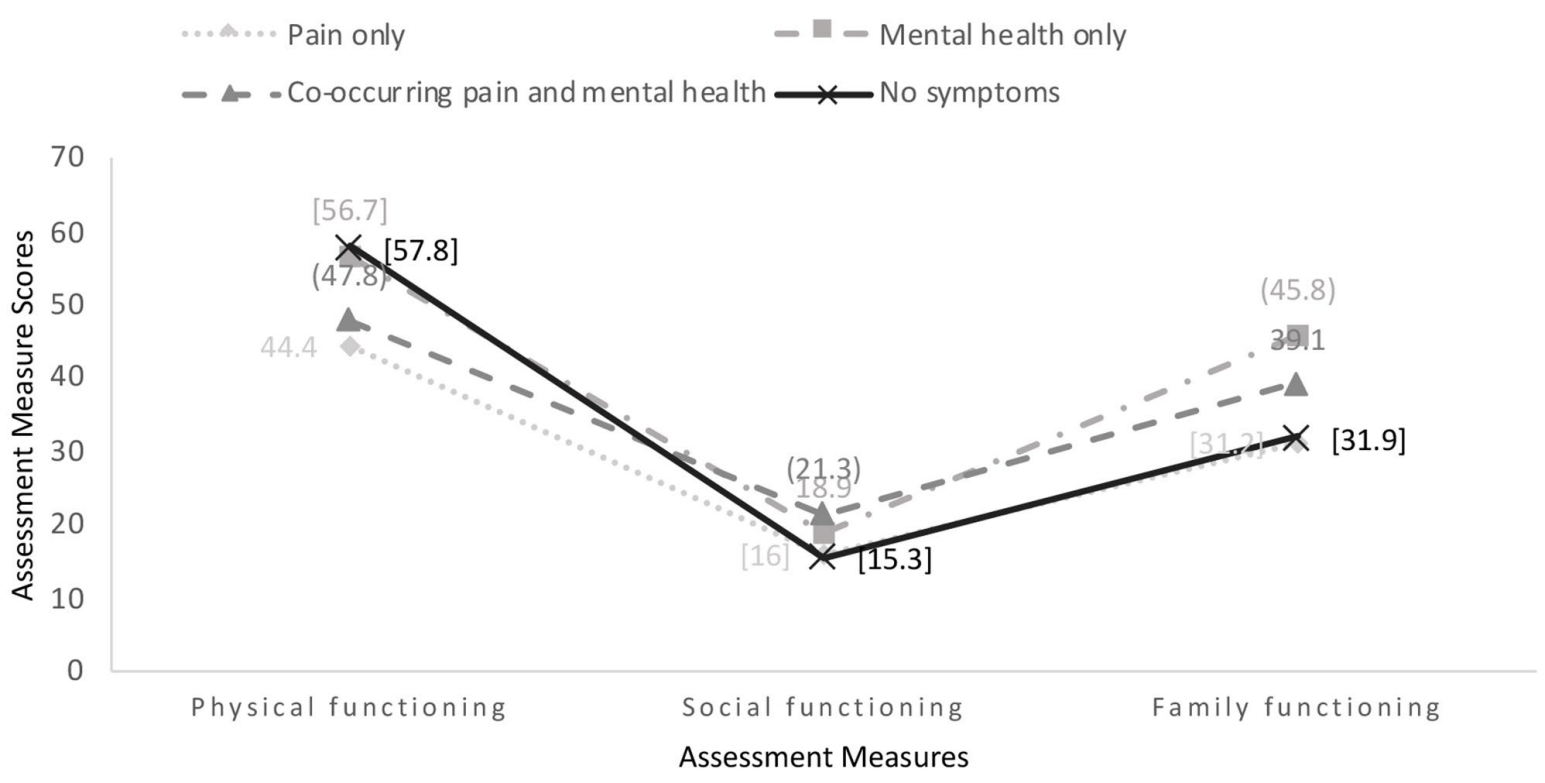
Significant functioning mean scores with pain intensity as a covariate. Note: Each shape represents the mean participant scores on the assessment measures for social, physical, and family functioning, for each of the groups. Higher scores for social and family functioning indicate more impaired functioning. Higher scores for physical functioning indicate better functioning. Scores marked with () represent statistically significantly differences than those in groups marked with []

An additional post hoc MANOVA was conducted that included pain intensity as a covariate and revealed the four groups were statistically different in their pain intensity scores (see Table 3). Thus, we included pain intensity as a covariate to a multivariate analysis of covariance (MANCOVA). Two participants elected not to provide pain intensity scores; thus, the results below are provided for *n* = 135 participants. Results revealed that the mean differences between the groups remained statistically significant, *F* (12, 387) = 4.15 *p* = .000, partial eta squared = 0.11 when controlling for pain intensity. Specifically, after controlling for pain intensity, we found significant differences between the groups for social functioning (*F* = (3, 130) = 7.32, *p* = .000, partial eta squared = 0.15) and family functioning (*F* = (3, 130) = 8.73, *p* = .000, partial eta squared = 0.17). Further analyses revealed that social functioning scores differed between adolescents with co-occurring pain and mental health symptoms with adolescents with pain-only (mean difference = 5.25, SE = 1.46, *p = .*003), and adolescents with no symptoms (mean difference = 6.03. SE 1.78, *p* = .006) on the other hand. Family functioning scores also differed significantly between adolescents with mental health-only symptoms with adolescents with pain-only symptoms (mean difference = 14.63, SE = 4.01, *p* = .002) and adolescents with no symptoms on the other hand (mean difference = 13.96, SE = 3.38, *p* = .000, see Fig. 1).

### Physical functioning mobility analysis

The PROMIS physical functioning mobility scores were analysed using a one-way between-groups analysis of variance (ANOVA). As the variance across the groups was not equal, we used a more stringent significance level of 0.01. The analysis revealed that the difference between the physical functioning mobility mean scores were statistically significant among the four groups (*F* = (3, 133) = 62.3, *p* = .000, partial eta squared = 0.13), see Table 3 for means. The effect size, calculated using eta squared, was 0.58. Further analysis using Tukey’s Honestly Significant Difference test (HSD) revealed the mean scores were significantly different for adolescents with co-occurring pain and mental health symptoms compared to those adolescents with mental health-only symptoms (mean difference = -14.14, SE = 1.3) and adolescents with no symptoms (mean difference = -16.30, SE = 1.7). Specifically, adolescents with co-occurring pain and mental health symptoms report worse physical functioning mobility than those with mental health-only symptoms and those with no symptoms. Likewise, adolescents with pain-only symptoms were statistically different from adolescents with mental health-only symptoms (mean difference = -15.81, SE = 1.8) and adolescents with no symptoms (mean difference = -17.96, SE = 2.1): those with pain-only symptoms reported the lowest scores and worse physical functioning mobility than those with mental health-only symptoms and those with no symptoms.

### The influence of Pain Intensity on Physical Functioning

Pain intensity was added as a covariate to analysis of covariance (ANCOVA) to determine the role of pain intensity on the scores of the physical functioning mobility measure (see Table 3). Results for *n* = 135 participants, who provided pain intensity scores, showed that the difference in physical function mobility remained statistically significant (F = (3, 128) = 17.14, *p* ≤ .001, partial eta squared = 0.29) when controlling for pain intensity. Pairwise comparisons show that the differences remained significant for adolescents with co-occurring chronic pain and mental health symptoms when compared to adolescents with mental health-only symptoms (mean difference = -12.91, SE = 1.9) and those with no symptoms (mean difference = -13.61, SE = 2.3). Specifically, adolescents with co-occurring chronic pain and mental health symptoms report worse physical functioning mobility than their peers with mental health-only symptoms, when controlling for pain intensity. Also remaining statistically significant were the scores between adolescents with pain-only symptoms versus those with mental health-only symptoms (mean difference = -12.60, SE = 2.3) and those with no symptoms (mean difference = -13.30, SE = 2.4, see Fig. 1), meaning that adolescents with pain-only symptoms report worse physical function mobility than adolescents with mental health-only symptoms and those with no symptoms.

## Discussion

Our study findings identified that adolescents with co-occurring pain and mental health symptoms as well as those with pain-only symptoms reported significantly worse physical functioning mobility than adolescents with mental health-only symptoms and those without symptoms. Significantly worse social functioning was reported by adolescents who experienced co-occurring chronic pain and mental health symptoms compared with adolescents who experienced pain-only symptoms and those with no symptoms. Lastly, adolescents with mental health-only symptoms experienced significantly worse family functioning than their peers with pain-only symptoms and no symptoms. In sum, the combined impact of pain and mental health symptoms was only found for the social functioning domain.

Research has consistently shown that adolescents who experience chronic pain and heightened levels of mental health symptoms (irrespective of whether a diagnosis has been received or not) report impaired physical and social functioning [16, 31]. Our research adds to growing evidence by showing that, when controlling for pain intensity, social functioning only was significantly worse for adolescents who experienced both chronic pain and mental health symptoms when compared with peers with pain-only or no symptoms. This suggests that adolescents with co-occurring chronic pain and mental health symptoms may feel more isolated than their peers in our other groups. Indeed, evidence highlights how adolescents who experience chronic pain frequently report feeling isolated [12, 32]. Interestingly, we found no significant differences between our groups when evaluating peer relationships, suggesting that whilst adolescents in our study reported challenges to social functioning generally, they did not feel the same way about the quality of their friendships. This finding is in contrast to findings of work conducted by Kashikar-Zuck et al. [33] which showed that adolescents with chronic pain were less well-liked, were less often picked as a best friend, and found to have fewer reciprocated friendships than adolescents without chronic pain. Consequently, whilst the adolescents in our study perceived themselves to have no significant peer relationship challenges, it is possible that their peers may perceive such peer related challenges to exist. It may introduce another perspective in the complex aspects of social functioning of adolescents with chronic pain and highlights the importance of conducting detailed multi-informant assessments of adolescents’ social functioning.

Another explanation for the reported challenges to social functioning, as compared to other functioning domains in this study, in our group with co-occurring pain and mental health symptoms may be the simultaneous burden of physical discomfort and psychological distress. This combination of symptoms can be overwhelming, reducing the adolescents’ ability to participate in social interactions [34]. The presence of mental health symptoms may contribute to social challenges. For example, Kingery et al. found that anxiety or depression can impair social skills, making it difficult for adolescents to initiate and maintain conversations, understand social cues, or build relationships [35].

In contrast to existing research [16, 31], our study found that no increase in physical functioning impairment was reported by adolescents with both mental health and chronic pain symptoms compared with adolescents reporting only chronic pain symptoms. One explanation for the difference in our study findings may be the predominently musculoskeletal nature of pain complaints in our sample, which may lead to greater difficulties with physical functioning overall regardless of mental health symptoms.

Another possible explanation for this difference could be due to the diverse mental health symptoms experienced by our participants. Previous studies have focused on distinctive mental health symptoms for example anxiety [31], rather than the range of conditions that were reported by our study participants. Across this wide range of mental health symptoms, the considerable impact pain symptoms have on physcial functioning might be too overpowering to allow for an additive impact of co-occuring mental health symptoms. Additionally, this divergence in our results may be due to the varying ways in which mental health symptoms are assessed and reported across adolescent research, with many studies, including this study, relying on high scores on assessment measures. Use of high scores on assessment measures in place of providing a diagnostic clinical interview to participants is typical across research. Although undoubtably more time consuming, use of diagnostic clinical interviews would faciliate a deeper understanding, and more comprehensive representation of the diversity and severity of both conditions and faciliatate comparison across studies.

Regarding family functioning, adolescents in the mental health-only symptoms cohort reported significantly lower levels of family functioning compared with the other groups. This was an interesting result as previous research has shown that worse family functioning is associated with the experience of living with a mental health disorder [36]. No causal relationship could be determined from the Sadler study, so the authors determined that the existence of a mental health disorder may contribute to poor family functioning, but equally, difficulties surrounding family functioning may also contribute to the onset of a mental health disorder [36]. While our findings further support the literature on poorer family functioning in adolescents with mental health symptoms, there was no significant differences in family functioning for our cohort of adolescents with co-occurring symptoms. A potential explanation for this finding is that those adolescents who experience chronic pain may often require additional support and depend on their parents more than their peers who do not live with pain [37]. Such reliance on parents may extend beyond meeting physical needs to those of emotional needs, potenitally subsequently resulting in a closer parent-adolescent bond, mitigating the difficulties seen in the mental health-only cohort.

### Strengths and limitations

A strength of our study includes recruitment of participants with a wide range of mental health symptoms compared with typical studies that focus on depressive and/or anxiety related symptoms only. We acknowledge possible limitations of our research due to the Covid-19 pandemic as adolescent behaviour and functioning were potentially affected by the closure of clinics and schools with subsequent Covid-19 related research revealing adverse mental health and feelings of lonliness [38] and depression and anxiety [39] for the adolescent population. Consequently, this unprecedented impact on adolescent’s lives may have impacted responses provided by adolescents in this study and reduced the ability to identify differences between the groups. Additionally, symptoms such as suicidal thoughts, autistic traits, perfectionism, poor sleep and stress appeared uncharacteristically low in our sample. This might be because adolescents listed symptoms experienced for three months or more in a free text box, focussing on the most prevalent symptoms experienced during the pandemic. Another limitation of using self-report methods to assess mental health symptoms and functioning in our participants is the lack of corroboration in the adolescents’ assessments of their symptoms. We therefore recommend that any future research should include assessments from multiple informants, e.g. adolescents, their parents, or if relevant clinicians, to provide a more comprehensive evaluation of adolescent mental health and functioning. Furthermore, the grouping of participants based on their self-reported symptoms of mental health and chronic pain differs from dimensional self-report tools, such as the Revised Children’s Anxiety and Depression Scale (RCADS), which provide a more comprehensive assessment of symptom severity across multiple domains. Whilst practical, for our participants without a formal diagnosis, our approach may lack the depth and diagnostic rigor of structured clinical interviews with psychotherapists, which are necessary for assessing full-blown clinical diagnoses and comorbidities. A further limitation of our study was our lack of assessment of social media use. Understanding adolescent’s social media use may impact on the perception of adolescents’ social functioning [40]. An additional limitation of our research is the majority of white participants within our sample, limiting generalisation of our findings. We acknowledge the importance of recruiting a more representative sample in future work and direct readers to the three part anti-racism in pain research by [41] to faciliate their understanding of how disregarding racism in pain research slows the research field and perpetuates pain inequalities.

There are important clinical implications that stem from our findings. Firstly, our social and physical functioning results highlight the need for standardised assessment measures for chronic pain and mental health symptoms to be developed to assess the mulidimensional nature of co-occurring symptoms. The current available resources offer a wide-range of methods and concepts for consideration, however they rarely allow for a wide variety of mental health disorders or diverse functioning domains to be assessed, thus, mitigating a true understanding of the co-occurrence of chronic pain and mental health symptoms. We have highlighted the disparity between our social functioning and peer relationship findings, revealing that there are clear differences between these domains. It is important to disentangle the exact challenges experienced by adolescents who are experiencing co-occurring chronic pain and mental health symptoms to enable a comprehensive assessment of functioning and a tailored treatment programme to be developed and stimulate integration of mental health and physical health care programmes. For instance, our results highlight the need for addressing the negative impact of social functioning, particularly for adolescents with co-occuring symptoms. Within therapy, it may be important for clinicians to particularly explore social functioning and to address this as part of the work where adolescents identify that they would like this to be different. Even in the face of challenges imposed by living a life with chronic pain, improving social functioning may help to reduce mental health symptoms and improve well-being, and subsequently, health related quality of life. A recent paper outlined how the absence of protective social relationships faced by people living with chronic pain can be a source of toxic stress [42]. Future research needs to continue to explore the additive impact of a wide range of mental health symptoms in depth and to explore these patterns over time to determine both causality and change with therapeutic input. Such an approach will ensure that a more accurate picture of the additive impact on functioning and associated needs is captured.

## Conclusion

Adolescents with co-occurring pain and mental health symptoms experience worse social functioning compared to individuals living with pain-only, as well as those reporting no pain or mental health symptoms. Social functioning is at the heart of adolescence and this influence may have considerable lifelong impact. Thus, we suggest that standardised and comprehensive assessment should be developed for adolescents experiencing chronic pain and mental health symptoms. Assessment measures should aim to assess a wider variety of mental health disorders and span diverse functioning domains to allow for a thorough understanding of co-occuring symptoms presented by young people in order to appropriately inform individualised, interdisciplinary treatment plans.

## Electronic supplementary material

Below is the link to the electronic supplementary material.

Supplementary Material 1

## Data Availability

Data analysed during this study are included in this published article [and its supplementary information files]. The raw datasets generated and/or analysed during the current study are not publicly available due to [consent to share was not obtained from individuals for ethical approval].

## Abbreviations

ADHD: Attention deficit hyperactivity disorder
CAMHS: Child and Adolescent Mental Health Services
BAPQ: The Bath Adolescent Pain Questionnaire
PROMIS: The Patient Reported Outcomes Measurement Information System
SCORE-15: The Systemic Clinical Outcome and Routine Evaluation
MANOVA: Multivariate analysis of variance analyses
ANOVA: Analysis of variance
MANCOVA: Multivariate analysis of covariance analyses
ANCOVA: Analysis of covariance
CRPS: Complex Regional Pain Syndrome
HSD: Honestly Significant Difference test (19)
