# Supplementary material for: The impact of co-occurring chronic pain and mental health symptoms on adolescent functioning, a cross-sectional survey
Source: BMC Psychol. 2024 Nov 6;12:629. doi: 10.1186/s40359-024-02126-5 (PMC11539307; doi:10.1186/s40359-024-02126-5)
Supplement: Supplementary file 1 — Supplementary Material 1 [file 40359_2024_2126_MOESM1_ESM.docx]

| **Supplementary Material**  **Table S1**  *Demographic and Condition Related Characteristics of the Sample* | | |
| --- | --- | --- |
| Characteristic | Number (%) | SD |
| Mean age range 11-19 | 16.07 | 1.8 |
| Ethnicity |  |  |
| White | 131 (95.6) |  |
| Black | 1 (0.7) |  |
| Asian | 2 (1.5) |  |
| Mixed | 3 (2.2) |  |
| unknown | 1 (0.7) |  |
| Gender |  |  |
| Male | 17 (12.4) |  |
| Female | 114 (83.2) |  |
| Non-binary | 6 (4.4) |  |
| Category |  |  |
| Pain | 20 (14.6) |  |
| Mental health | 45 (32.8) |  |
| Both pain and mental health | 54 (39.4) |  |
| No symptoms | 19 (13.9) |  |
| Recruitment method |  |  |
| friend‎/family (snowballing) | 27 (19.7) |  |
| Social media | 45 (32.8) |  |
| Pain clinic | 33(24.0) |  |
| Online pain charity | 4 (2.9) |  |
| School/college/university | 28 (20.4) |  |
| Sites pain experienced in |  |  |
| Lower limbs | 38 (27.7) |  |
| Back pain | 34 (24.8) |  |
| Headache/ migraine | 30 (21.9) |  |
| Multisite pain | 26 (19.0) |  |
| Neck pain | 24 (17.5) |  |
| Upper limbs | 23 (16.8) |  |
| Abdominal pain | 20 (14.6) |  |
| Muscle pain | 21 (15.3) |  |
| Whole body pain | 4 (2.9) |  |
| Chest pain | 2 (1.5) |  |
| Ankle/foot pain | 1 (0.7) |  |
| Uterus pain | 1 (0.7) |  |
| Rib pain | 1 (0.7) |  |
| Soles of foot pain | 1 (0.7) |  |
| Joint pain | 1 (0.7) |  |
| Facial pain | 1 (0.7) |  |
| Jaw pain | 1 (0.7) |  |
| Other pain | 14 (10.2) |  |
| Duration of chronic pain symptoms |  |  |
| One year and under | 10 (7.3) |  |
| 1<2 years | 12 (8.8) |  |
| 2<3 years | 15 (10.9) |  |
| 3-4 years | 8 (5.8) |  |
| 4<5 years | 4 (2.9) |  |
| Over 5 years | 25 (18.2) |  |
| Diagnosed pain condition | 49 (35.7) |  |
| CRPS | 22 (16.1) |  |
| Chronic pain disorder | 14 (10.2) |  |
| Migraine/headache | 4 (2.9) |  |
| Scoliosis | 4 (2.9) |  |
| Hypermobility | 3 (2.2) |  |
| Chronic Fatigue | 2 (1.5) |  |
| Fibromyalgia | 2 (1.5) |  |
| Abdominal pain | 1 (0.7) |  |
| Pulmonary stenosis | 1 (0.7) |  |
| Dystonia | 1 (0.7) |  |
| Period pain | 1 (0.7) |  |
| Allodynia | 1 (0.7) |  |
| Other | 5 (3.6) |  |
| Mental health symptoms experienced |  |  |
| Anxiety | 68 (49.6) |  |
| Depression | 41 (29.9) |  |
| Low mood | 24 (17.5) |  |
| Panic attacks | 16 (11.7) |  |
| Self-harm | 10 (7.3) |  |
| Disordered eating | 9 (6.6) |  |
| Low self esteem | 6 (4.4) |  |
| Negative or intrusive thoughts | 6 (4.4) |  |
| Suicidal thoughts | 6 (4.4) |  |
| Lack of focus or motivation | 6 (4.4) |  |
| OCD | 5 (3.6) |  |
| PTSD | 4 (2.9) |  |
| Paranoia | 4 (2.9) |  |
| Anger | 4 (2.9) |  |
| Fatigue | 4 (2.9) |  |
| Audio and/or visual hallucinations | 3 (2.2) |  |
| Fluctuating moods | 3 (2.2) |  |
| Suppressed emotions or numbness | 3 (2.2) |  |
| Emotional dysregulation | 2 (1.5) |  |
| Manic episodes | 2 (1.5) |  |
| Autistic traits | 2 (1.5) |  |
| Agitation | 1 (0.7) |  |
| Alexithymia | 1 (0.7) |  |
| Skin Picking | 1 (0.7) |  |
| Dissociation | 1 (0.7) |  |
| Perfectionism | 1 (0.7) |  |
| Excessive worry | 1 (0.7) |  |
| Phobia | 1 (0.7) |  |
| Frustration | 1 (0.7) |  |
| Stress | 1 (0.7) |  |
| Rumination | 1 (0.7) |  |
| Poor sleep | 1 (0.7) |  |
| Self-isolation | 1 (0.7) |  |
| Substance misuse | 1 (0.7) |  |
| Premenstrual dysphoric disorder | 1 (0.7) |  |
| Loneliness | 1 (0.7) |  |
| Isolation | 1 (0.7) |  |
| Impulsive behaviours | 1 (0.7) |  |
| Other mental health symptoms | 2 (1.5) |  |
| Duration of mental health symptoms |  |  |
| One year and under | 17 (12.4) |  |
| 1<2 years | 11 (8.0) |  |
| 2<3 years | 17 (12.4) |  |
| 3-4 years | 10 (7.3) |  |
| 4<5 years | 8 (5.8) |  |
| Over 5 years | 31 (22.6) |  |
| Diagnosed mental health condition | 55 (40.1) |  |
| Anxiety disorder | 37 (27.0) |  |
| Depression | 24 (17.5) |  |
| Mixed anxiety | 2 (1.5) |  |
| PTSD | 10 (7.3) |  |
| OCD | 5 (3.6) |  |
| Anorexia | 4 (2.9) |  |
| Eating disorder or disordered eating | 3 (2.2) |  |
| Major depressive disorder | 3 (2.2) |  |
| Social anxiety disorder | 3 (2.2) |  |
| Autism spectrum disorder | 2 (1.5) |  |
| Anger issues | 2 (1.5) |  |
| Panic disorder | 2 (1.5) |  |
| Attachment Disorder | 1 (0.7) |  |
| Bulimia | 1 (0.7) |  |
| Emotional dysregulation | 1 (0.7) |  |
| Trichotillomania | 1 (0.7) |  |
| Low mood | 1 (0.7) |  |
|  |  |  |
